# The functional role of integrins during intra- and extravasation within the metastatic cascade

**DOI:** 10.1186/s12943-018-0937-3

**Published:** 2019-01-18

**Authors:** Greta Sökeland, Udo Schumacher

**Affiliations:** Institute of Anatomy and Experimental Morphology, University Cancer Center, University Medical Center Hamburg Eppendorf, Martinistraße 52, 20246 Hamburg, Germany

**Keywords:** Cancer, Epithelial mesenchymal transition, Selectin, Integrin, Integrin ligands, Leukocyte adhesion cascade, Metastasis, Extravasation, Prognosis, Integrin inhibitor

## Abstract

Formation of distant metastases is by far the most common cause of cancer-related deaths. The process of metastasis formation is complex, and within this complex process the formation of migratory cells, the so called epithelial mesenchymal transition (EMT), which enables cancer cells to break loose from the primary tumor mass and to enter the bloodstream, is of particular importance. To break loose from the primary cancer, cancer cells have to down-regulate the cell-to-cell adhesion molecuIes (CAMs) which keep them attached to neighboring cancer cells. In contrast to this downregulation of CAMS in the primary tumor, cancer cells up-regulate other types of CAMs, that enable them to attach to the endothelium in the organ of the future metastasis. During EMT, the expression of cell-to-cell and cell-to-matrix adhesion molecules and their down- and upregulation is therefore critical for metastasis formation. Tumor cells mimic leukocytes to enable transmigration of the endothelial barrier at the metastatic site. The attachment of leukocytes/cancer cells to the endothelium are mediated by several CAMs different from those at the site of the primary tumor. These CAMs and their ligands are organized in a sequential row, the leukocyte adhesion cascade. In this adhesion process, integrins and their ligands are centrally involved in the molecular interactions governing the transmigration. This review discusses the integrin expression patterns found on primary tumor cells and studies whether their expression correlates with tumor progression, metastatic capacity and prognosis. Simultaneously, further possible, but so far unclearly characterized, alternative adhesion molecules and/or ligands, will be considered and emerging therapeutic possibilities reviewed.

## Background

### General steps of the metastatic cascade

**Fig. 1:**
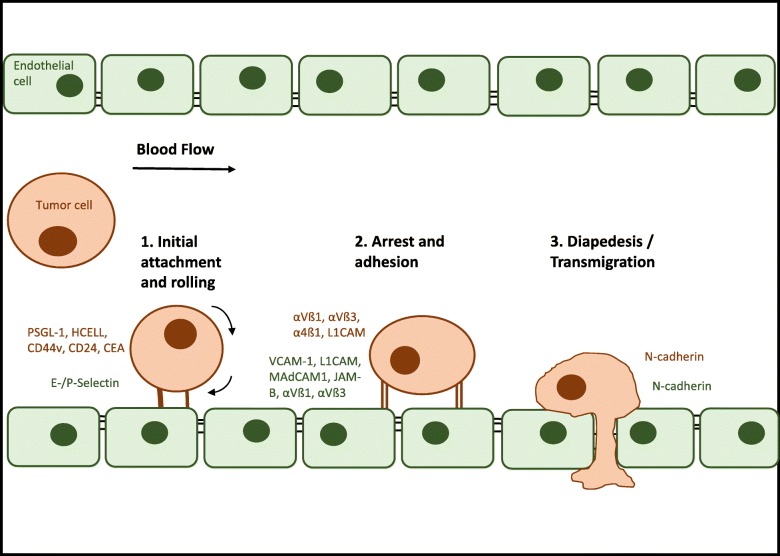
The extravasation of tumor cells. To achieve improved clarity the figure is limited to the major adhesion molecules and their interactions. Tumor adhesion molecules are shown in brown, endothelial ligands are shown in green

The capacity for metastatic dissemination as the ultimate attribute of malignancy is acquired during malignant progression. Vogelstein and Kinzler summarize this evolution towards malignancy as “Three Strikes to Cancer”. Initially, a driver-gene mutation unleashing abnormal proliferation represents the first strike in the pathway to cancer. A second driver-gene mutation then initiates the expansion phase. This mutation enables the cell to thrive in its local environment and adapt to low-growth factor concentrations, oxygen, nutrients and functioning cell-to-cell contacts. After the first two strikes, cancer cells still satisfy criteria for benignity as they do not metastasize. The last strike driving the invasive phase brings on the malignant character of cancer, enabling it to invade surrounding tissues and disseminate through the body. However, despite considerable research efforts, a genetic signature for metastasis formation has not been identified [1]. The first step of metastasis formation consists in neoplastic cells loosening themselves from the primary tumor cell mass and breaking down the basement membrane of the tumor blood vessels, allowing stroma invasion and intravasation. The second step is for the cells to survive transport through the circulation, and as a third step, to arrest at the luminal side of the normal blood vessel endothelium in a distant organ (see Fig. 1). After transmigration of the endothelial barrier (fourth step), the cells have to adapt to the new microenvironment and have to commence proliferation (fifth step) [2]. The process by which the cancer cells gain migratory and invasive properties is called the epithelial-mesenchymal transition (EMT) [2]. Normal epithelial cells, from which cancer cells arise, are closely bound to their neighboring epithelial cells. This form of tissue organization is achieved through the sequential arrangement of adherens junctions, desmosomes and tight junctions [3]. The EMT program involves downregulation of cell-to-cell and cell-to-matrix adhesion molecules, dissolution of adherens and tight junctions and a loss of cell polarity, to overcome the natural barrier and become motile [2]. Additionally, mesenchymal cell adhesion molecules are upregulated and expressed on the cell surface, creating invasive cells with both a mesenchymal and a stem cell-like phenotype, enabling dissemination [3]. At the metastatic site this transition is reversed by the process of mesenchymal-epithelial transition (MET). This conversion to a more epithelial cell phenotype embodies an important factor in the formation of macrometastasis and metastatic colonization [3]. These findings suggest that transformation of the cancer cell adhesion molecule pattern may play the key role in metastatic spread.

This review focuses on the role of integrins and other adhesion molecules for tumor cell extravasation in metastatic dissemination (see Fig. 1). It examines whether mesenchymal adhesion molecules and/or the expression of their ligands on cancer cells correlates with tumor progression, metastatic capacity and prognosis. Additionally, their value as prognostic markers and their potential as oncologic treatment targets will be discussed.

### Extravasation of leukocytes and tumor cells

Extravasation constitutes a multistep phenomenon that can be divided into different phases. The extravasation process is initialized by rolling, relatively low-affinity binding, of leukocytes and/or tumor cells mediated by the selectin family of adhesion molecules (see Fig. 1). Rolling is followed by tight adhesion through integrins and other adhesion molecules. After adhesion, leukocytes and tumor cells transmigrate through the vascular endothelium by a procedure named diapedesis. Leukocytes are able to transmigrate following a paracellular or transcellular route driven by adhesion molecule interactions [4]. In contrast to leukocytes, presumably cancer cells do not leave the endothelium intact after diapedesis. This seems reasonable if one keeps in mind the size of tumor cells in comparison to leukocytes [4]. One of the few mechanisms known for tumor cell diapedesis lies in N-cadherin interactions [5] (Fig. 1). This insufficiency in knowledge of means for diapedesis of tumor cells should be subject for further examination. The complete process of extravasation is also known as the leukocyte adhesion cascade as its steps have to be followed in a sequential order. However, some redundancy within the molecules involved in this cascade may be possible. In leukocytes, this cascade is a physiological process they use to migrate to inflammatory sites. In contrast, tumor cells take advantage of this mechanism in hematogenous metastasis to disperse through the blood circulation in the entire body [4] (see Fig. 1).

### Selectin mediated rolling

The cells move to the margin of the blood stream (margination) where rolling is initiated by selectins, lectin cell adhesion molecules, expressed on the surface of leukocytes and endothelial cells [4] (see Fig. 1). The selectins are one of the smallest human gene families, consisting of three members only, namely E(ndothelial)-, P(latelet)- and L(ymphocyte) selectin. E- and P-selectins expressed by endothelial cells are of particular importance for the extravasation of leukocytes at inflammation sites and for the purpose of metastasis formation [6]. E-selectin (CD62E) is expressed in the first hours of immune-inflammatory reactions, mediating the interaction with leukocytes (e. g. neutrophils and lymphocytes). E-selectin is synthesized de novo by cytokine-activated (e. g. IL-1, TNFα or interferon-ɣ) endothelial cells [7]. In addition, E-selectins are constitutively expressed on skin and bone microvascular endothelium and in the vasculature of the bronchial mucosa [6]. They represent binding partners for PSGL-1 (P-selectin glycoprotein ligand-1) and CLA (cutaneous lymphocyte antigen, decorated PGSL-1) found on monocytes, neutrophils and lymphocytes [6]. Additionally, HCELL (hematopoietic cell E−/L-selectin ligand) [8], found on stem cells, and CD34 act as a E-selectin ligand. P-selectin (CD62P) is pre-synthesized and stored in Weibel-Palade bodies of the endothelium and α-granules of platelets [6]. Pro-inflammatory mediators such as cytokines and histamine lead to an instant expression at the luminal surface of endothelial cells [7, 9].

**Fig. 2:**
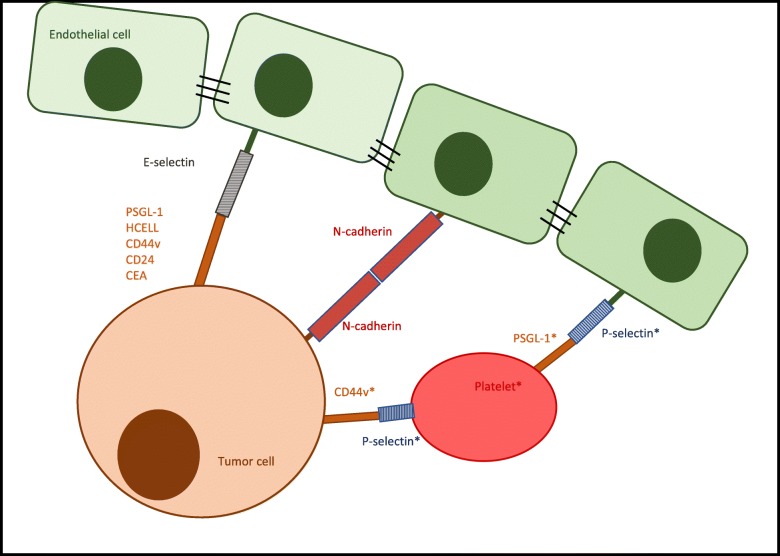
Rolling of tumor cells. Representation of tumor cell rolling mediated by the interaction with selectin adhesion molecules. (*) Example of a possible interaction between tumor cell and the endothelium using an intermediate to link both interaction partners, forming a bridge [6, 156]

Tumor cells exploit these mechanisms used by leukocytes to roll, arrest and adhere to the vascular endothelium [6, 10, 11] (see Fig. 2). The extravasation of colon cancer cells is regulated by the activation of E-selectin on the endothelial layer. By means of a p38- and ERK-dependent signaling pathway the integrity of the endothelium is modified, contributing to tumor cell extravasation [12]. LS174T colon carcinoma cell lines make use of the CD44 glycoform also known as hematopoietic cell E−/L-selectin ligand (HCELL) to interact with E- and L-selectin [13]. Furthermore, other variant isoforms of CD44 (CD44v) on LS174T colon cancer cells retain P-, L-, E-selectin binding activity [14]. Similar E-selectin interactions have been seen in other colon carcinoma cell lines [15, 16]. Moreover the carcinoembryonic antigen (CEA) has been identified as a potential binding partner for E- and L-selectin [17]. Prostate cancer cells express glycoprotein and glycosphingolipid structures containing sialyl Lewis X epitopes to adhere to E-selectin on bone marrow endothelial cells, promoting metastatic dissemination into the bone [18]. PSGL-1 containing sialyl Lewis X epitopes, have been identified as one ligand for E-selectin in prostate cancer cells [19]. Additionally, CD24 expressed on breast cancer cells acts as an alternative interaction partner for E-selectin [20]. In conclusion, E-selectin acts as a homing receptor in the hematogenous dissemination of breast cancer [21], lung adenocarcinoma [22], prostate cancer [18], colon cancer [13] and pancreatic carcinoma cells [23]. P-selectins expressed by platelets may also adhere to selectin ligands on tumor cells or mucin fragments released by neoplastic cells, forming a bridge between the carcinoma cells, the platelets and the inflamed vasculature (see Fig. 2) [6].

Less importance in metastatic spread has been attributed to L-selectins. In contrast to E- and P-selectins, L-selectins (CD62L) are not expressed by endothelial cells. Instead, they are found on the surface of lymphocytes and other types of leukocytes, such as neutrophils and monocytes [24]. They mediate rolling on high endothelial venules, by binding to constitutively expressed ligands, summarized as peripheral lymph node addressins [4]. Similar to P-selectins, L-selectins might allow the formation of a bridge between tumor cells and the endothelium through a leukocyte (instead of a platelet) as an intermediate [6] (see Fig. 2). Data collected from Läubli et al. suggests that L-selectin-interactions of leukocytes at the sites of embolization may enhance tumor cell extravasation [25]. Lastly, N-cadherin has been observed to play a minor role in the regulation of rolling and adhesion of for eg. MDA-MB-468 human breast carcinoma cells by the means of a N-cadherin/N-cadherin interaction [4, 26] (see Fig. 2).

### Integrin mediated adhesion

**Fig. 3:**
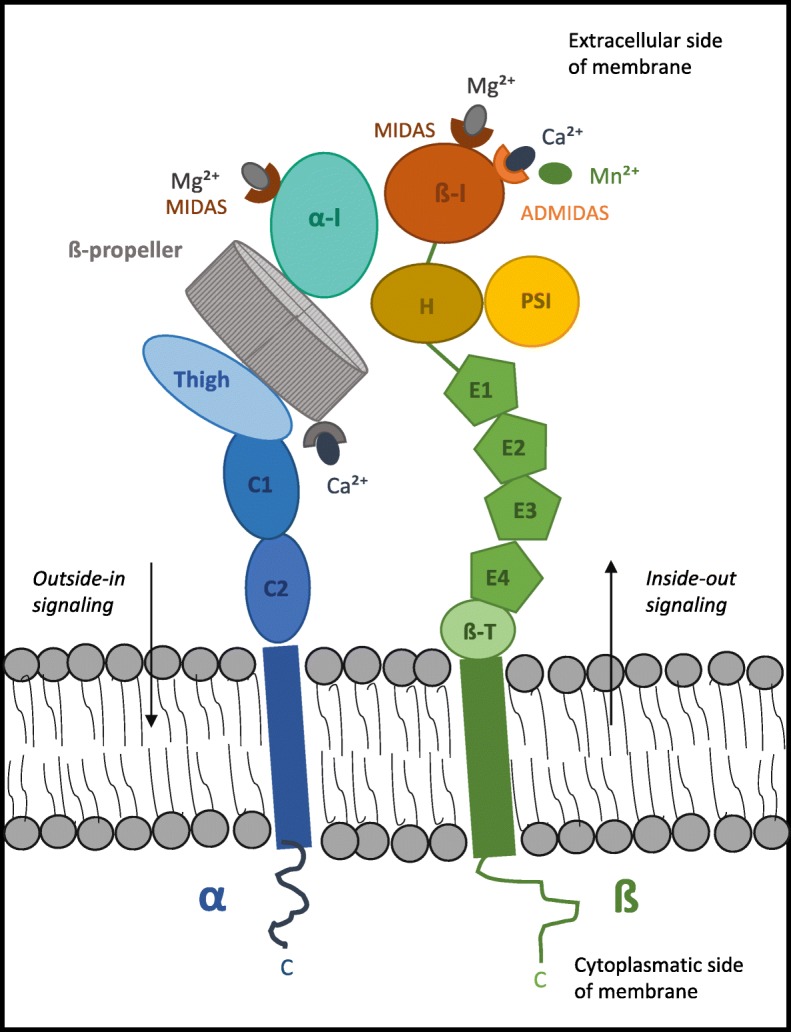
Representation of an αI-domain-containing integrin heterodimer and the distribution of its domains. All integrins contain a ßI domain in the ß subunit, whilst nine out of the 18 integrin α chains contain an αI-domain of around 200 amino acids, inserted between blades two and three of the ß-propeller [35, 157]. The α subunit is formed by a calf-2 (C2), calf-1 (C1) and a thigh domain supporting the seven-bladed ß-propeller [31]. The αI-domain is coordinated by a Mg^2+^ ion in the metal-ion-dependent adhesion site (MIDAS) and represents the ligand-binding site. The ß subunit contains a MIDAS and ADMIDAS site mediating conformational changes resulting in an active form of the integrin. The ß subunit contains a ßI-domain, a hybrid domain (H), a plexin-semaphorin-integrin (PSI), four cysteine-rich epidermal growth factor repeats (E1–4) and a ß-tail/transmembrane (ß-T) domain [35]. As bi-directional signaling receptors, integrins convey inside-out and outside-in signaling [31]

During this selectin-mediated rolling, integrins are activated, bind to their endothelial ligands and permit the tight adhesion of leukocytes [4]. Integrins are a large family of homologous transmembrane cell adhesion proteins that tie the matrix to the cytoskeleton or serve as cell-to-cell adhesion molecules [27] (see Fig. 3). The connection between integrins and the cytoskeleton is mediated by the integrin adhesome network, also known as integrin adhesion complex (IAC) [28]. Integrins consist of two non-covalently associated transmembrane glycoprotein subunits described as α and ß. The α subunit determines integrin-ligand specificity, whilst the ß subunit is connected to the cytoskeleton and affects different signaling pathways [29]. In vertebrates, 18 α and 8 ß subunits have been described that can assemble into 24 heterodimers [30, 31]. Nine of the 18 α chains contain an αI domain consisting of an approximately 190–200 amino acid residue sequence near the N terminus of the integrin α subunit [32, 33]. This domain is found in the ß2 integrin subgroup, αEß7 and in the collagen-binding ß1 integrins (α1, α2, α10 and α11) [29]. Ligand-binding depends on a coordinating Mg^2+^ ion in the metal-ion-dependent adhesion site (MIDAS) motif in the αI domain bridging ligand binding [32]. The ß subunit contains a ßI domain with an Mg^2+^ coordinating MIDAS and a region adjacent to it (ADMIDAS), binding an inhibitory Ca^2+^ ion [31]. The active form of the integrin is achieved by Mn^2+^ ions binding to the ADMIDAS site and thereby inducing a conformational change [31]. The ßI domain plays a role in ligand binding in non-αI containing integrins [29] (see Fig. 3).

Ligand recognition seems to be mediated by conformational changes dictating active and inactive states of integrins [34]. The general mechanisms of ligand recognition are thought to be alterations in the tertiary conformation of the αI domains deciding upon the activation state of ß2 integrins; quaternary structural changes likely also play a role [34]. Recognition is further brought about by signaling generated by the assembly of complexes, the adhesome network, on the cytoplasmatic side of the membrane [35]. Activation of leukocytes can arise from “inside-out signaling” triggered by agonists binding to different receptors, thus allowing ligand recognition by ß2 integrins, causing changes in affinity and avidity. In addition, the involvement of integrins conveys “outside-in signaling”, giving rise to intracellular transduction cascades that mediate functional responses and the integration with other signals [34] (see Fig. 3). Due to the fact that integrins lack enzymatic activity, signaling is instead induced by the arrangement of signaling complexes on the cytoplasmatic side of the plasma membrane [35]. The assembly is reached about by receptor clustering and by activating conformational changes that generate or expose binding sites [35].

**Fig. 4:**
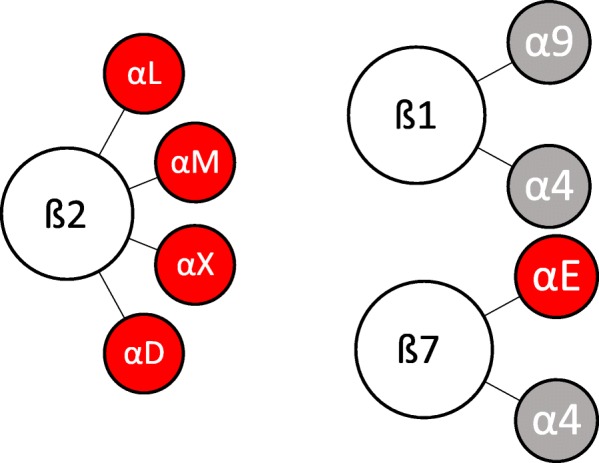
Leukocyte lineage integrins. All of the leukocyte integrins [29] bind to LDV motifs. Subunits containing an αI-domain are marked in red

A key characteristic of most integrins is their capability to bind to a wide repertoire of ligands. Undeniably, the expanse of the integrin family is outrun by their number of ligands [27]. Determinants of the integrin-ligand interaction are the affinity and the conformational state of the integrin and the availability and conformational state of the ligand. These characteristics dictate the choice of any given integrin to interact with one of their multiple ligands. To further add to the complexity of the interactions, integrin ligands, that is to say other adhesion molecules and extracellular matrix proteins, bind to multiple integrins and have no unique specific binding partner [27, 36]. Nevertheless, it is possible to divide integrin-ligand combinations according to their underlying structure, driving molecular interaction into four distinct classes, keeping in mind that other additional interactions exist [37]. This division may be a starting point at which to systematize the complex network of integrin interactions and to understand the integrin patterns tumor cells may use to imitate leukocyte extravasation. Humphries et al. differentiated between RGD-binding integrins: αV, ß1 (α5, α8) and αIIbß3, recognizing ligands with an RGD active site. The RGD motif (tripeptide Arg-Gly-Asp) interacts at an interface between α and ß subunits. The R residue binding with a ß-propeller module and the D coordinating a cation bound in the ßI domain [37]. In contrast, LDV- binding integrins (α4ß1, α4ß7, α9ß1, ß2 subfamily, αEß7) interact with LDV motifs (Leu-Asp-Val) on their ligands and can contain αI domains (see Fig. 4). Additional classes are represented by the A-domain (αI domain) (α1, α2, α10, α11) ß1 integrins binding to laminin and collagen and the Non αA-domain-containing laminin-binding integrins (α3ß1, α6ß1, α7ß1, α6ß4) [37].

The ß2 and ß7 heterodimers are restricted to cells of the leukocyte lineage [29] (see Fig. 4). Each class of leukocytes expresses a specific pattern of integrins. A common characteristic is that every leukocyte subtype expresses at least a single member of the ß2 integrin family [34]. Integrin α4ß1 is expressed on monocytes, on neutrophils in the case of sepsicemia [38] and on T cells. In T cells the integrin associated protein CD47 regulates adhesion of the ß2-integrins VLA-4 (α4ß1), as well as LFA-1. Integrin VLA-4 is additionally expressed on immune globulin G-producing plasma cells [39]. Instead of expressing VLA-4, B cells synthesizing IgA antibodies make use of integrin α4ß7 to migrate to mucosal tissues [39, 40]. Furthermore, α4ß7 integrin is expressed on homing T cells playing a role in the ability to migrate to skin and gut [40]. Memory T cells express α4ß1 and LFA-1 for trafficking [41]. All integrins expressed on leukocytes, including α4ß1, α9ß1, αLß2, αMß2, αXß2, αDß2, αEß7 and α4ß7 integrins, represent LDV-binding integrins [37] (see Fig. 4).

Although leukocytes express specific integrins for the interaction with ligands during extravasation, these leukocyte-integrins could be substituted on tumor cells by other integrins or adhesion molecules with the ability to bind to the same ligands on the vascular endothelium. This is due to the fact that a wide range of ligands may bind to a single integrin and vice versa. Thereupon, tumor cells wouldn’t mimic leukocytes by expressing identical integrin patterns but by expressing integrins and adhesion molecules with receptors for the same ligands on the vascular endothelium. It appears that the binding pattern of leukocytes and tumor cells could be identical, despite the fact that their integrins may differ. Furthermore, tumor cells possess alternative ways of extravasation, such as the use of leukocytes as linker cells [4].

## Integrins expressed on leukocytes and tumor cells and their ligands

### Integrin αLß2 (LFA-1) and adhesion molecule ICAM-1

The adhesion of leukocytes is achieved by different types of integrins. Integrin αLß2 (lymphocyte function associated antigen LFA-1) acts as a ligand for ICAM-1 (intercellular adhesion molecule 1, CD54) [42] ICAM-2 (CD102) [43] and the junctional adhesion molecule A (JAM-A, member of immunoglobulin superfamily) [44]. Furthermore, the integrin αMß2 (Mac-1) can bind to ICAM-1/− 2 and JAM-C on the endothelium [45, 46]. ß2-Integrin LFA-1 is expressed on neutrophils and lymphocytes, whereas Mac-1 is mainly found on neutrophils [39]. Tumor cells do not express such integrins; nevertheless, they possess the ability to express ICAM-1 and use leukocytes as linker cells to adhere to the vascular endothelium [4](Fig. 4). In this way, they adhere to the vascular endothelium by means of an ICAM-1/LFA-1 interaction with the leukocyte, which for its part binds to ICAM-1 on the endothelium through LFA-1.

**Fig. 5:**
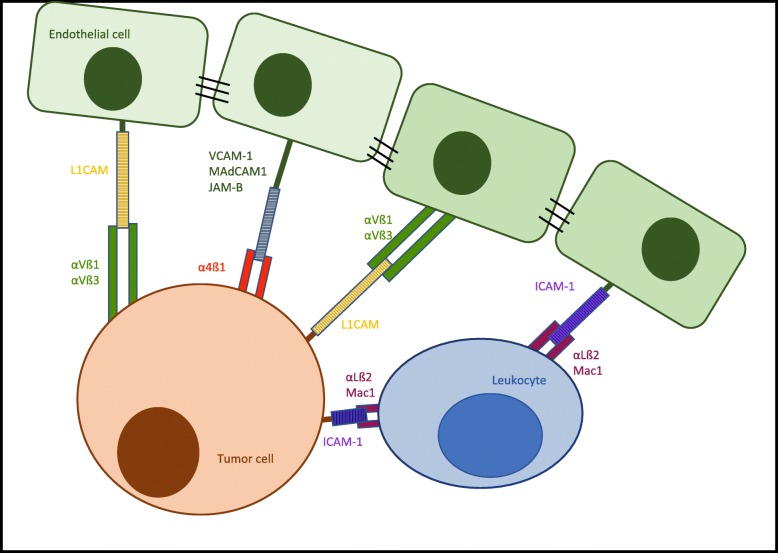
Tumor cell adhesion molecules. Representation of cell adhesion mediated by integrins and other cell adhesion molecules binding to their corresponding endothelial ligands. Depiction of the specific integrins that are found overexpressed on tumor cells allowing extravasation and formation of distant metastases

The genes on chromosome 19p13.2 encoding intercellular adhesion molecules (ICAM) have been determined as a cancer susceptibility locus [47, 48]. In vitro targeting of ICAM1 reduced breast cancer cell invasion and metastasis [48]. Furthermore, ICAM1 expression correlated with the metastatic capacity of five human breast cancer cell lines, suggesting its key role in invasion and dissemination [48]. It has been shown that MDA-MB-468 human breast carcinoma cells use leukocytes as linker cells to adhere to lung endothelial cells [26]. Being ß2-integrin negative, they express ICAM-1, which interacts with αMß2 or αLß2 on neutrophil granulocytes. The neutrophil granulocytes then, via αMß2 or αLß2, bind to the L1-CAM-positive endothelial cells [26] (Fig. 5). The use of leukocytes as linker cells for adhesion and transmigration has further been observed in melanoma cell metastatic dissemination [49]. Melanoma cells bind to ß2-integrins on polymorphonuclear neutrophils (PMN) via ICAM-1, whereas PMN themselves bind to ICAM-1 present on the endothelium [49]. ICAM-1 is further expressed in oral squamous cell carcinoma (SCC). Analysis has revealed that it is predominantly found at the invasive front of oral squamous carcinoma and its expression correlates with the level of invasion and lymph node dissemination [50]. Its expression is also associated with increased angiogenic and lymphangiogenic activity [50]. ICAM-1 increases the metastatic capacity of esophageal squamous cell carcinoma cells (ESCC) and stimulates tumorigenesis in a mouse model [51]. It further enhances sphere formation and therefore raises resistance to radio- and chemotherapy. These cancer characteristics have partly been attributed to a p53-dependent pathway [51].

Additionally, ICAM-1 upregulation is found in aggressive papillary thyroid carcinoma cells. Expression levels correlate with tumor malignancy, angiolymphatic invasion and metastasis [52]. Poorly differentiated thyroid carcinomas show higher ICAM-1 expression levels than well-differentiated carcinomas, supporting the thesis that ICAM-1 upregulation induces a more aggressive tumor phenotype [52]. Similar findings in investigations of ICAM-1 expression in salivary adenoid cystic carcinoma accentuate the role in hematogenous metastasis. Salivary adenoid cystic carcinoma cells use leukocytes as a bridge to attach to the endothelium by means of ICAM-1. Expression levels correlate with lymph node invasion and local recurrence [53]. ICAM-1 expression was expressed in half of a gastric patient collective (49.0% of 108 gastric cancer patients), and its expression levels were associated with a more advanced stage of gastric cancer and lymph node metastasis [54]. Higher ICAM-1expression levels were detected in most (90.9%) of the gastric cancer patients with liver metastasis [54]. Patients with non-small-cell lung cancer have been shown to have higher serum levels of sICAM-1 (soluble ICAM-1) and increased ICAM-1 expression. Rising slCAM-1 levels predicted a short-term fatal outcome, whilst total levels had no prognostic value [55].

ICAM-1 is important in the metastatic spread of osteosarcoma cancer cells. It has been shown that TGFα in osteosarcoma increases ICAM-1 expression levels, transforming cancer cells into a more motile, invasive and adhesive phenotype [56]. Likewise, in prostate cancer, bradykinin, which has been shown to promote tumor expansion and dissemination, enhances migration and spread by augmenting ICAM-1 expression [57]. A study by Belal Al-Husein et al. revealed that micrometastasis in prostate cancer could be inhibited by blocking integrin αVß3 and ICAM-1, thus implying its function in overcoming the endothelial barrier and allowing hematogenous metastasis [58]. In addition, αVß3 integrin is essential for prostate cancer cell migration and adherence to bone matrix [59].

ICAM-1 expression has been observed in a heterogenous group of cancer entities described above, such as breast cancer [48], esophageal squamous cell carcinoma (ESCC) [51] and non-small-cell lung cancer [55] (see Table 2). In all these entities high ICAM-1 expression levels were associated with a more invasive, aggressive and motile phenotype allowing metastatic dissemination. Findings in ESCC even suggest that ICAM-1 expression may contribute to resistance against radio- and chemotherapy [51]. These findings and first in vitro studies on ICAM-1 expression or ICAM-1 regulatory pathway inhibition bring to mind its potential for targeted therapy and future prevention of metastasis [48, 58]. It still needs to be further determined whether the height of ICAM-1 levels could have prognostic value and if the levels have a clinical relevance and could be used for risk stratification to foresee adverse outcomes in oncologic patients.

The fact that integrins αDß2, αMß2 and αXß2 also show interaction with ICAM-1 makes them possible alternative binding partners that tumor cells could make use of to accomplish adhesion [37] (Table 2). It remains to be determined whether αDß2 and αMß2 are expressed on cancer cells. First studies on αXß2 (CD11c) expression in tumors do not suggest an involvement in metastasis and even seem to indicate a favorable prognosis in gastric cancer [60]. Nevertheless, this is due to the fact that αXß2 is expressed on tumor-infiltrating dendritic cells that mediate an anti-tumor immune response [60]. In this analysis, no expression of αXß2 on tumor cells was shown, and therefore, the assumption that tumor cells make use of αXß2 as an alternative ligand for ICAM-1 still needs to be further investigated.

### Integrin α4ß1 (VLA-4) and adhesion molecule VCAM-1

In the case of septicemia, neutrophil granulocytes express α4ß1 (very late antigen 4, VLA-4) on their surface [38], acting as a ligand for VCAM-1 (vascular cellular adhesion molecule 1, CD106), which is upregulated in response to inflammatory mediators. Similarly, the major integrin mediating firm adhesion in monocytes is VLA-4 (see Table 1). It is also expressed by lymphocytes granting adhesive functions [39]. Apart from attaching to VCAM-1, the junctional adhesion molecule B (JAM-B) represents an alternative binding partner [45]. In tumor cells, α4ß1 and α4ß7 integrins serve as alternative ligands for VCAM-1 and have been associated with the metastatic capacity of cancer [4, 61, 62] (see Fig. 5) (see Table 2). Additionally, VCAM-1, itself expressed on cancer cells, has been determined as a driver of metastatic dissemination due its competence to bind to α4ß1 found on the endothelium in lymph nodes [63].

**Table 1:** Overview of adhesion molecules found on different leukocyte subtypes and their corresponding ligands

Leukocyte Adhesion Molecule	Leukocyte Subtype	Ligand
VLA-4 (α4ß1)	Monocytes [39] Neutrophils in case of sepsis [38]	VCAM-1 MAdCAM-1 JAM-B
LFA-1 (αLß2) (CD11a/CD18)	Neutrophils [39, 42] Monocytes [43]	ICAM-1 ICAM-2 JAM-A
α4ß7 (CD49d/ß7)	Lymphocytes (CD4 and CD8 T cells) (mucosal lymphoid nodules) [7, 44]	MAdCAM-1
Gp150,95 (αXß2) (CD11c/CD18)	Monocytes Macrophages [45] Granulocytes [46] (Neutrophils [9]) Langerhans cells	iC3b (JAM-C [47])
αMß2 (mac-1) (CD11b/CD18)	Monocytes [43] Macrophages [45] Granulocytes [48] (Neutrophils [42]) Langerhans cells [7]	iC3b ICAM-1 ICAM-2 JAM-C
αDß2	Macrophages (?) [7] Neutrophils [9]	ICAM-3
CD47	T cell [49]	VCAM-1 ICAM-1
L1-CAM	Lymphocytes [4, 50, 51]	α5ß1 (VLA-5) αVß3
Glycosylated proteins (ß-galactose)	Leukocytes [52]	Galectins

Only major ligands are listed

**Table 2:** Integrins expressed on leukocytes and tumor cells and their ligands

Adhesion molecule	Family	Leukocyte subtype	Ligand	Expression on tumor cells
**VLA-4 (α4ß1)** **(CD49d)**	Integrin	Monocytes Neutrophils in case of sepsis	VCAM-1 MAdCAM-1 JAM-B	Melanoma, osteosarcoma, kidney carcinoma, leukemia, oral squamous cell carcinoma, ovarian cancer
LFA-1 (αLß2) (CD11a/CD18)	Integrin	Neutrophils Monocytes	ICAM-1 ICAM-2 JAM-A	
Gp150,95 (CD11c/CD18)	Integrin	Monocytes Macrophages Granulocytes Langerhans cells	iC3b JAM-C	
α4ß7 (CD49d/ß7)	Integrin	Lymphocytes (mucosal lymphoid nodules)	MAdCAM-1	
αMß2 (mac-1) (CD11b/CD18)	Integrin	Monocytes Macrophages Granulocytes Langerhans cells	iC3b ICAM-1 ICAM-2 JAM-C	
αDß2	Integrin	Macrophages ?	ICAM-3	
CD47		T cell	VCAM-1 ICAM-1	
**ICAM-1**	Ig superfamily	Monocytes Epithelial cells Fibroblasts	LFA-1 (leukocytes) Mac-1 (leukocytes)*	Breast cancer, melanoma, oral squamous carcinoma, esophageal squamous carcinoma, papillary thyroid carcinoma, salivary adenoid cystic carcinoma, gastric cancer, non-small-cell lung cancer, osteosarcoma, prostate cancer
ICAM-2 ICAM-3	Ig superfamily	Endothelial cells Leukocytes Langerhans cells	LFA-1 (CD11a)	
VCAM-1	Ig superfamily	Activated endothelial cells	VLA-4	
PECAM-1	Ig superfamily	Endothelial cells Monocytes Neutrophils	PECAM-1 (CD31)	
MAdCAM-1	Ig superfamily	Endothelial cells	L-selectin α4ß7	
**L1-CAM**	Ig superfamily	Tumor cells	L1-CAM α5ß1 αVß3 (endothelium)	Colorectal carcinoma, epithelial ovarian carcinoma, renal cell carcinoma, melanoma, pancreatic ductal adenocarcinoma, breast cancer
**αVß3**	Integrin	Tumor cells	L1-CAM	Prostate cancer, melanoma
**α5ß1**	Integrin	Tumor cells	L1-CAM	Breast cancer

CAMs in bold, have been identified on tumor cells (for references see text and table 1). *Leukocytes act as a bridge between tumor cell and the endothelium

High levels of integrin α4ß1 have been identified on A375M melanoma cells, allowing them to adhere to VCAM-1 on the vascular endothelium. In a mouse model, pulmonary metastasis formation could be stimulated by enhancing α4ß1 expression [61]. Likewise, lung colonization in B16-BL6 melanoma was increased by TNFα administration mediating higher levels of α4ß1 expression [62]. Brain metastatic tumor cells have shown high levels of α4ß1 integrin expression, suggesting a key role in metastatic seeding. Further, it has been shown that blocking overexpressed α4ß1 significantly diminishes the number of metastatic colonies within the brain, implying its potential as a therapeutic target [64]. In vitro assays have shown the involvement of α4ß1 integrin cell adhesion to the vascular endothelium in melanoma, osteosarcoma and kidney cancer cells [65]. Its overexpression has further been detected in leukemia cells and lymphoma cells [66]. The expression of integrin α4ß1 by lymphoma cells may be partially responsive for metastasis of lymphomas to the bone marrow where marrow stomal cells express VCAM-1 [66, 67]. In myeloma cells, myeloma cell adhesion to the bone marrow has been observed to be an α4ß1 integrin-dependent process [68]. Moreover, it seems that α4ß1 expression may be a marker of poor prognosis in children with acute lymphoblastic leukaemia and may play an important role in the response to therapy [69]. It still needs to be determined whether it could be used as a therapeutic target.

In addition, oral squamous cell carcinoma cells make use of integrin α4ß1 to attach to endothelial cells in the metastatic process [70]. In ovarian cancer α4ß1 plays a role in mesothelial invasion [71], mesothelial invasion being an unfavorable prognostic marker [72]. α4ß1 as a therapeutic target was analyzed in a study by Scalici et al. [73]. Treatment of ovarian tumor-bearing mice with anti-α4ß1 antibodies alone had no effect, whilst combined treatment with carboplatin, human-specific α4ß1 blocking antibodies and anti-VCAM-1 antibody significantly reduced the tumor burden [73]. The involvement of integrin α4ß1 has also been observed in growth-factor and tumor-induced lymphangiogenesis promoting metastasis [74]. As it plays a part in lymphangiogenesis and metastasis, blocking this integrin may be useful in preventing metastatic spread. Moreover, integrin α4ß1 expression correlates with radiotherapy resistance in head and neck cancer [75]. It further mediates resistance to Lapatinib in breast cancer [76] and Erlotinib resistance in lung cancer cells [77].

In conclusion, the above-named results make imply that VLA-4 participates in the metastatic cascade by binding to VCAM-1 on the vascular endothelium (see Fig. 5), and its inhibition therefore may constitute a possible key element for the prevention of metastatic spread. However, it remains to be determined whether it could be used as a prognostic marker. Integrins α9ß1 and αDß2 have been described as alternative interaction partners for VCAM-1 [37] (see Table 3). It remains uncertain whether they are overexpressed on tumor cells, and this may be subject to analysis in the future.

**Table 3:** Selection of integrin inhibitors in preclinical studies and clinical trials

Integrin Inhibitor	Target Integrin	Clinical trial
Intetumumab (CNTO 95)	αV	Phase II [129]
Abituzimab (DI17E6, EMD 525797)	αV	Phase I/II [153]
MK-0429	αVß3	–
Cilengitide (EMD 121974)	αVß3 αVß5	Phase II/III [135, 137]
D-pinitol	αVß3	–
GLPG0187	αVß3	Phase I [144]
Volociximab (M200)	α5ß1	Phase II [148]
PF-04605412	α5ß1	Phase I [149]

### Integrin α5ß1 (VLA-5) and adhesion molecule L1-CAM

Integrin α5ß1 (VLA-5), as well as αVß3 expressed on the vascular endothelium, interact with L1-CAM (neuronal cell adhesion molecule), a member of the immunoglobulin superfamily expressed on immune and neural cells [78–82]. With respect to its biological function, it has a static role as an adhesion molecule in cell-to-cell interaction and a motility-supporting role that drives cell migration during the development of the nervous system, and it also promotes metastatic dissemination [83–86] (see Fig. 5). It is a transmembrane glycoprotein of the immunoglobulin superfamily that can interact with different binding partners heterophilically (eg. to integrins, other neural cell adhesion molecules, CD24 etc.), or even homophilically to itself [87]. Work over the past years has shown the significance of L1-CAM as a pro-metastatic factor (see Table 2). It is overexpressed in many different types of malignancies, such as colorectal carcinoma [88], melanoma [89], renal clear cell carcinoma [90], pancreatic ductal adenocarcinoma (PDAC) [85], breast cancer [88], ovarian and endometrial carcinoma [91]. Dissimilar to its function in neural development, L1-CAM expression in cancer induces a motile and invasive tumor phenotype, promoting metastatic dissemination, aggressive tumor expansion and chemoresistance associated with a poor prognostic outcome [87]. It seldom acts as a stimulus in cell-to-cell adhesion, as in neural development [87]. These attributes associated to L1-CAM expression will be illustrated subsequently. For cancer cells to become motile, the functional mode of L1-CAM needs to be altered. This is brought about by two mechanisms: the cleavage from the cell surface by membrane proximal proteolysis and the capacity to change ligands and get involved in L1-CAM integrin interactions. In tumor cell lines, cleavage of the membrane proximal L1-CAM ectodomain is mediated by disintegrin and metalloproteinases (ADAMs) [87, 92]. Neoplastic cells cut homophilic adhesion molecules, converting them into mobile cells, thus allowing metastatic spread [93]. In addition, ectodomain cleavage appears to play a role in L1-CAM-mediated gene regulation of various cancer-related genes, which can promote a pro-tumorigenic and an anti-apoptotic gene expression pattern. Furthermore, the soluble ectodomain itself is functionally active stimulating cell migration [94], inhibiting apoptosis [95] and promoting angiogenesis [87, 96]. On the one hand, motility is encouraged by the soluble L1-CAM ectodomain after cleavage, and on the other hand, by over expression of L1-CAM-promoting migration by enhancing L1-CAM-ß1 integrin interactions [87]. Apart from α5ß1, L1-CAM interacts with RGD-binding integrins αVß5, αVß1, αVß3 and αIIbß3 [87]. As a binding partner for L1-CAM, α5ß1 Integrin (VLA-5) is allegedly found on the vascular endothelium and functions as an adhesion molecule in tumor cell extravasation [80]. One may assume that by expressing L1-CAM, the tumor cell may gain the key to a lock on its metastatic journey, allowing substantial progress, even if it may not remain the only lock to be opened on its way.

L1-CAM overexpression was first described in colon cancer cell lines with aberrantly activated ß-catenin-T-cell factor (TCF) signaling. The promoter of the L1-CAM gene is activated by the ß-catenin-TCF pathway [88, 89]. In addition, L1-CAM expression correlates with metalloprotease ADAM10 expression, driving L1-CAM to become functional [88]. A fact supporting the hypothesis of L1-CAM as a promoter of the induction of a motile tumorigenic phenotype is its known role in cell migration during the development of the nervous system. L1-CAM and ADAM10 have been found at the invasive front of colorectal cancers [88]. Furthermore, a study revealed L1-CAM expression in 13% of colorectal cancer patients who underwent surgical resection [97]. Analysis of L1-CAM expression unveiled a significant correlation of L1 expression, with the dissemination of tumor cells in lymph nodes and bone marrow. In addition, comparison of survival in L1-CAM-positive and -negative patients retrospectively revealed a significantly poorer outcome for positive L1-CAM patients; five-year overall survival was decreased by 20% [97]. Another study determined L1 expression in colorectal carcinoma to be 10.9%, and it was significantly associated with distant metastasis [98]. Additionally, L1-CAM expressing colon cancer cells, when injected into the spleen of mice, promote the spread of tumor cells forming liver metastasis [99]. These findings suggest that L1-CAM expression may have a prognostic value in colorectal carcinoma, and further exploration is required into whether it may be used as a prognostic marker.

L1-CAM is upregulated in ovarian and uterine carcinomas, and its expression is associated with adverse outcome in terms of short survival [91]. In these tumor entities, first results suggest that L1-CAM expression may be used as a prognostic marker, and it could be of aid to identify patients with uterine cancer who are at high risk for recurrent disease [91]. L1-CAM expression patterns in epithelial ovarian carcinoma have been analyzed. The normal ovarian surface epithelium shows a specific expression pattern of L1-CAM, whilst the highly invasive epithelial ovarian carcinoma cells display an enriched pattern, suggesting its role in neoplastic transformation [100]. Furthermore, expression was significantly associated with poor outcome and a more invasive cancer phenotype. It has been shown that L1-CAM plays a dual role in ovarian surface epithelial cells, supporting a tumor-suppressive function in non-transformed cells on one hand; and on the other hand promoting invasion in ovarian cancer cells by the means of a functional-switch and an increase in its expression [100]. These results imply that L1-CAM may represent a therapeutic target and should therefore be explored. L1-CAM overexpression has been found in 46% of clear cell renal carcinoma and in 28% of papillary renal cell carcinoma [89]. Expression significantly correlated with the Ki-67 proliferation index [89]. In renal clear cell carcinoma, its presence was further associated with metastatic spread, the risk being greater if cyclin D was not expressed in the cancer cells [90]. The L1-CAM+/cyclin D- profile was found to be a prognostic factor for the presence of metastasis [90].

Additionally, 42% of malignant melanoma tissues show L1-CAM presence [89]. Its expression has been associated with sustained activation of the extracellular signal-regulated kinase (ERK) pathway, resulting in an upregulation of gene products such as αVß3 integrin, thus mediating a motile tumor phenotype [101]. The correlation of L1-CAM expression with melanoma progression and migration is significant [101], and upregulation has been associated with metastasis [102]. Overexpression further induces conversion of the radial melanoma growth phase to the vertical growth phase, representing a more invasive phenotype [101]. L1-CAM function suppression significantly reduces invasive growth, but it did not completely block melanoma invasion and migration. Meier et al. additionally analyzed different function-blocking antibodies of L1. Their findings acknowledge L1-CAM as an important player in melanoma invasion and progression, and they recognized its therapeutic potential, especially in combination with conventional cancer treatment, although blockage of L1 alone may not be enough to cease or reverse invasive growth [101]. In pancreatic ductal adenocarcinoma (PDAC), L1-CAM expression was found in 80% of patients [85], while other studies showed focal L1-CAM expression in 2% [103]. Expression seems to be involved in anti-apoptotic protection and chemoresistance. The transformation to a chemoresistant pancreatic ductal adenocarcinoma phenotype involves an increased IL-ß-dependent secretion of nitric oxide (NO) and the engagement of L1-CAM. High IL-ß-dependent L1-CAM expression conferred anti-apoptotic protection to cancer therapy agents in neoplastic cells [85]. Apart from PDAC, L1-CAM overexpression is linked to a more motile phenotype of breast cancer cells [89]. On one hand, L1-CAM expression is downregulated by Nm23-H1 (metastasis suppressor gene) mediating non-metastatic breast cancer cells; on the other hand, cells lacking Nm23-H1 had high L1-CAM levels and a more motile phenotype [104].

We have seen that L1-CAM expression is detected in various cancer entities (see Table 2) and its expression most significantly correlated with tumor progression, invasion and metastatic spread. It has also been associated with chemoresistance [85] and may be used as a prognostic marker in some cases [90, 91]. As a key player in the metastatic pathway of different tumors, its value as a potential therapeutic target becomes more and more evident.

### Integrins αVß3 and α5ß1

Apart from L1-CAM expression being observed on tumor cells, the expression of its ligands αVß3 and α5ß1 has been observed [105, 106] (see Fig. 5). These integrins bind to L1-CAM found on endothelial cells and mediate a more invasive tumor cell phenotype. A non-neuronal form of L1-CAM is also found on epithelial cells [107]. Integrin α5ß1, as an interaction partner for L1-CAM, has been discovered to be implicated in glycoprotein nmb-mediated (GPNMB) breast cancer progression [106] (see Table 2). In contrast, integrin αVß3, representing a binding partner for L1-CAM, is expressed on various types of tumor cells. The integrin αVß3 is involved in angiogenesis, neovascularization and tumor metastasis [108] (see Table 2). Investigations of the expression of αVß3 on prostate cancer cells has been highly contradictory. It has been indicated that prostate cancer cells use αVß3 to stimulate angiogenesis and thereby facilitate growth and metastasis [105]. Furthermore, Cooper et al. attributed integrin αVß3 a role in the adhesion of prostate cancer cells to the endothelium and in the metastatic dissemination to the bone through a PI-3 kinase / Akt pathway [105]. Nevertheless, other investigations show that most prostate tumor cells are immunonegative for αVß3 [109, 110]. These controversial findings will need further clarification.

In addition, αVß3 has been found to be involved in the progression of the radial growth phase melanoma towards the invasive vertical growth phase melanoma [111, 112]. The expression is low or absent in the radial growth phase [113]. Once the tumor cells upregulate the ß3 integrin expression, tumor thickness increases and the melanoma cells gain the capacity to invade, spread and colonize distant organs [111, 113]. In a similar manner, the overexpression of α4ß1 integrin leads to the more invasive phenotype of melanoma tumor cells and seems to have an effect on the metastatic potential of melanoma cells [112, 114]. Essential for metastatic spread is transendothelial migration of the tumor cells; for this process melanoma cells make use of αVß3 integrin [113]. As stated earlier, the melanoma cells lacking αVß3 integrins can make use of ß1 integrins instead to adhere to the endothelial ligand VCAM-1 [112]. The integrin α4ß1 is only found on highly metastatic melanoma cell lines such as MV3 and BLM [115].

## Integrins as therapeutic targets

As illustrated above, carcinoma cells express numerous integrins mediating the adhesion to the vascular endothelium and thereby promoting extravasation and metastasis. Therefore, the pharmacological inhibition of these integrins could represent a way of slowing down or even stopping cancer progression.

Integrins represent a therapeutic target for antibody-based drugs (most abundant integrin targeting drugs), small-molecule-based drugs and peptide-based drugs [112]. Further, first data reveals that targeting integrins using chimeric antigen receptor-engineered T-cells might as well be of use in T cell immunotherapy of solid tumors [116]. In the last years several integrin-targeted drugs are showing promising results in preclinical studies, others have advanced to clinical trials and some have been approved for clinical use [117]. Nineteen of the 24 integrin heterodimers have been therapeutic targets for new pharmacological agents [118]. Presently, there are some pharmaceutics targeting αIIbß3 integrin to prevent platelet aggregation in percutaneous cardiac intervention, acute coronary syndromes and myocardial infarction, approved by the U.S. Food and Drug Administration (FDA) [117]. Furthermore, there is one FDA approved α4 antagonist named Natalizumab for the treatment of multiple sclerosis and Crohn’s disease, binding α4ß1 and α4ß7 [117]. To date, there are no FDA-approved αVß3 and αVß5 integrin antagonists as well as α5ß1 integrin antagonists, although some are under evaluation in clinical trials for the treatment of inflammatory and neoplastic diseases [117, 119].

Intetumumab (CNTO 95), a human anti-αV integrin antibody, inhibits melanoma cell adhesion, migration and invasion in vitro and decreases tumor growth in melanoma xenografts in mice [112, 120]. It is currently evaluated by a randomized, phase II study [121]. Orally active αVß3 integrin inhibitor MK-0429 reduced lung metastasis and melanoma burden in the mouse model [122]. MK-0429 was also evaluated in patients with hormone-refractory prostate cancer and bone metastasis [123]. It was generally well tolerated and there was some evidence of reduction of osteoclast activity, indicating a potential for clinical use in the treatment of hormone-refractory prostate cancer, eventhough serum prostatic specific antigen (PSA) was unexpectedly increased during the trial [123, 124]. A randomized phase II study of Etaracizumab, a monoclonal antibody against αVß3, ± dacarbazine in patients with stage IV metastatic melanoma, showed no improvement in survival over decarbazine alone [125] (see Table 3). Another selective inhibitor of the integrins αVß3 and αVß5 is Cilengitide (EMD 121974) [112, 126]. On one hand, a randomized phase II study in patients with metastatic melanoma showed good tolerance. On the other hand, Cilengitide only showed minimal clinical efficacy as monotherapy in metastatic melanoma [127]. In addition, Cilengitide is being evaluated for the treatment of other neoplastic diseases. In an osteosarcoma mouse model it inhibited pulmonary metastasis and minimally decreased primary tumor growth [128]. Its anti-angiogenic and anti-metastatic functions have also been assessed in a phase III clinical trial for glioblastoma multiforme therapy [129]. The lack of efficacy in this trial was a surprise and discouragement [130]. Although some studies are still ongoing and their results pending, up to date, all clinical trials evaluating αVß3 and αVß5 inhibitor Cilengitide, amongst others in squamous cell carcinoma of the head and neck, glioblastoma and non-small cell lung carcinoma, have failed their primary endpoints [118] (see Table 3). The overall results and the detailed revision of these trials will decide whether αVß3 and αVß5 integrin antagonists might yet become an effective therapy and make their way to clinical use in oncologic treatment.

Abituzimab (DI17E6, EMD525797), a humanized monoclonal antibody against integrins containing an αV-subunit, inhibited migration and invasion of prostate cancer cells in a preclinical model [131]. In a multicenter phase 1 trial in advanced prostate cancer with bone metastases there was evidence for clinical activity, which needs to be confirmed in the phase II trial [132]. Abituzumab is further investigated in metastatic colorectal cancer (phase I/II POSEIDON trial), although the primary end point was not reached, biomarker analyses revealed subgroups of patients whom may have benefited [133] (see Table 3). Further, integrin αVß3 inhibitors are being studied for the treatment of prostate cancer [124]. An analysis revealed anti-metastatic effects of D-pinitol, a phytochemical, in human prostate cancer cells by reducing the cell surface expression of αVß3 integrin. Further, D-pinitol modulated FAK, c-Src and NF-κB pathways leading to an added inhibition of αVß3 integrin [134]. Other preclinical models of prostate cancer demonstrated that the inhibition of αV-integrins, in particular αVß3 integrin, by GLPG0187, a broad spectrum integrin receptor antagonist, reduced de novo formation as well as progression of bone metastases in prostate cancer [135]. A phase I study of GLPG0187 in patients with advanced solid malignancies failed to demonstrate signs of single-agent therapy efficacy [136] (see Table 3). However, other experimental investigations suggest that αVß3 does not play an important role in the adhesion of prostate cancer cells and its blockage causes no observable effect on the number of adhered cells [137], which could explain the failure in efficacy of αVß3-antagonists in phase I and II trials. These controversial findings should be subject to further clarification. In the same analysis blocking α5ß1 integrin, instead of αVß3, reduced the number of adherent prostate cancer cells [137]. Similar to the results obtained in studies evaluating αVß3-antagonists, the inhibition of integrin αVß3 has had little therapeutic effect in several trials for solid tumors [118]. Raab-Westphal et al. suggest that the controversy between preclinical and clinical data may result partly from the fact that xenograft models do not reflect human tumors genetically, nor with respect to pharmacokinetic and pharmacodynamics behavior, well enough [118]. Furthermore, some data indicates that αVß3 and αVß5 integrins are not essential for angiogenesis and pathological vascular development apparently reversing the previously widely reported and suggested importance of the integrins for angiogenesis [138]. These conflicting results only highlight the need for further evaluation.

Volociximab (M200), a α5ß1 integrin inhibitor, has displayed promising results in preclinical and clinical trials [112, 139]. It is being evaluated for monotherapy and combined treatments in different tumor entities such as metastatic melanoma [112], ovarian and peritoneal cancer [140]. In a phase II study in patients with ovarian or peritoneal cancer Volociximab showed insufficient clinical activity [140] (see Table 3). A different α5ß1 integrin antagonist, integrin monoclonal antibody PF-04605412, demonstrated no anti-tumor activity too in a first-in-human clinical trial, leading to a discontinuation of its clinical development [141]. The N-terminus of the ß1-domain of integrin α5ß1 also represents a target for ATN-161, a small peptide, which inactivates the integrin [142]. In mice, the combined treatment with α5ß1-inhibitor ATN-161 and chemotherapeutic 5-fluorouracil (5-FU) diminished colorectal liver metastases, reduced angiogenesis and improved survival [142]. In a phase I trial of ATN-161 in patients with solid tumors, about 1/3 of the patients that administered ATN-161 displayed prolonged stable disease and different dose levels were well tolerated [143] (see Table 3). Some more recent preclinical studies of targeted chemotherapy of α5ß1 integrin positive solid tumors show promising antitumor effects in vitro and in vivo in mice, that appear encouraging for further investigation [144].

Although many preclinical trials show promising results, most clinical trials evaluating integrin inhibitors in neoplastic disease have failed to meet the expectations as anti-tumor agents. Nevertheless, the use of integrin-targeting drugs in thrombosis prevention after percutaneous coronary intervention (PCI) (αIIbß3 integrin), ulcerative colitis, Crohn’ disease and multiple sclerosis (α4ß1 and α4ß7 integrins) [145] show that integrins do represent a potential therapeutic target and may show clinical efficacy in metastases prevention henceforth. It may only be necessary for some reevaluation to take place to be able to overcome the gap between preclinical and clinical trial results and achieve the desired tumor control in the future. Hereafter some problems in relation to integrin inhibitors will be discussed.

Cells express multiple integrins and other adhesion molecules [146]. Integrins retain redundancy in ligand interaction and signaling functions, and there is extensive crosstalk between integrins affecting cell functions [147]. A form of integrin-crosstalk, trans-dominant inhibition of integrin activation, takes place when ligand binding to one integrin inhibits the activity of another integrin [147]. This implies that blocking one integrin may only lead to the upregulation of another related integrin, rather than inhibiting a complete signaling pathway, to maintain adhesion, interaction and signaling. Additionally, a highly selective antagonist of a single integrin may make the development of resistance or paradoxical effects more likely [146]. Sheldrake and Patterson have characterized the potential of dual and multi-integrin-antagonists as more efficient inhibitors of cancer progression and dissemination [146]. It seems plausible that the inhibition of a single integrin may not suppress the complete adhesive cell function in a cell that expresses a pattern of different integrins. Apart from dual and multi-integrin inhibitors, the combination of integrin antagonists with other drugs with anti-tumor activity such as chemotherapeutics are being evaluated [142, 148]. An integrin inhibitor could intensify and reinforce the anti-tumor effect of an oncologic treatment, rather than be used as a single-agent.

A different reason for the failure of single integrin inhibitors in stopping cancer cell dissemination and slowing down disease progression might be the fact that selectin interactions come first and are not altered by integrin antagonists. Apart from using integrin mediated adhesion, ovarian cancer cells predominantly employ selectin-dependent leukocyte-like adhesion cascades in peritoneal spread [149]. These cascades have also been observed in peritoneal metastasis of pancreatic adenocarcinoma [150]. These selectin-dependent cascades might not be altered at all or enough by integrin antagonists to prevent the extravasation of cancer cells.

How integrin inhibitors may interfere with the integrins physiological function, is another issue to be discussed and reviewed. Genetic and cell biological analysis has revealed a number of different integrin functions [151]. However, integrins do not simply act as glue-like molecules; they constitute bi-directional signaling receptors involved in outside-in and inside-out signaling [31, 152]. As cell surface receptors integrins coordinate the assembly of cytoskeletal polymers and signaling complexes on the cytoplasmatic face of the plasma membrane [37, 151]. On the extracellular face integrins interact with extracellular matrix substrates or counterreceptors on cell surfaces, allowing cell-to-matrix and cell-to-cell linkages [31]. These interactions are essential for cell migration by supplying traction to migrating cells and by transferring guidance signals that direct moving cells and their targets [151]. Cell migration being of great significance in developmental processes [153] and in cell extravasation during inflammatory response [4]. Further, cell-to-cell interaction, integrin-mediated adhesion, embodies a key role in tissue integrity and is of particular importance permitting tissues to withstand mechanical load [151]. Additionally, integrins contribute to tissue differentiation and organogenesis [151]. The abundancy of functions and occurrence of integrins implies distinct potential side effects their inhibition could involve. Nevertheless, one must keep in mind that various phase I studies focusing on toxicity of integrin inhibitors have shown good tolerance and no major adverse events [121, 127, 143].

## Conclusion

In conclusion, various integrins have been reported to be overexpressed in cancer cells, promoting a more motile and invasive phenotype (see Fig. 5, Table 2). It remains unknown whether tumor cells express an even greater range of adhesion molecules as has yet been described. The specification and exploration of possible alternative adhesion molecules on tumor cells for known ligands (see Table 4) may be of interest in the definitive and more detailed characterization of the tumor cell integrin expression pattern. This is due to the fact that tumor cells, even when imitating leukocyte mechanisms for extravasation, do not express the exact same adhesion molecule patterns as leukocytes. Table 4 shows alternative adhesion molecules tumor cells could express, instead of the integrins found on leukocytes, to bind to endothelium. A definitive characterization of the integrin expression pattern of tumor cells may give new insights and answers to the question why some integrin inhibitors aren’t showing the expected positive results in clinical trials. In addition, it may open up possibilities for combined integrin inhibitor therapies.

**Table 4:** Alternative adhesion molecules

Leukocyte Adhesion Molecule	Alternative Adhesion Molecule	Ligand
VLA-4 (α4ß1)	α4ß7 α9ß1 αDß2	VCAM-1
VLA-4 (α4ß1)	α4ß7	MAdCAM-1
α4ß7	α4ß1	MAdCAM-1
LFA-1 (αLß2)	αXß2 αMß2 αDß2	ICAM
Gp150,95	αXß2 αMß2	iC3b
L1-CAM	αVß1^a^ αIIBß3^a^	α5ß1 αVß3

^a^αVß1 and αIIBß3 as possible alternative adhesion molecules bind to L1-CAM and therefore could only be of use for tumor cells to bind to leukocytes mediating adhesion to the endothelium as linker cells

The table shows alternative adhesion molecules (Humphries et al. 2006) tumor cells could use instead of the adhesion molecules present on leukocytes to adhere to the vascular endothelium for extravasation

In many tumor entities, high levels of integrin/integrin ligand expression correlate with cancer progression, invasion and metastasis. First studies suggest that integrin expression patterns may have prognostic value and that they may be used to identify patients at high risk of adverse outcome in risk stratification [91]. Nevertheless, sufficient evidence still needs to be revealed. Apart from having a prognostic value, they might be of use in imaging. Integrins represent a target for selective imaging agents that might allow detection of metastases [154, 155]. Principally, they represent a potential therapeutic target and their blockade could be a way of preventing distant organ seeding. To understand the implications the discovery of a new therapeutic alternative could have, one must keep in mind that metastatic dissemination constitutes one of the major challenges in oncologic treatment. In many patient histories, metastases transform a disease considered curative into a palliatively approached one. The variety of integrins found upregulated on tumor cells gives cause to doubt whether blocking a single integrin could represent a promising way of stopping metastatic spread. It is more likely that to achieve the desired effect, whole integrin patterns will have to be inhibited to minimize extravasation, the phenotypic transformation and angiogenesis. Furthermore, it needs to be elaborated in greater detail to what extent such a blockade would interfere with the integrins’ physiological function in the body, such as leukocyte extravasation or any other cell-to-cell or cell-to-matrix interaction [27]. Nevertheless, once the gap between reassuring preclinical studies [101, 122] and the corresponding less encouraging clinical trials has been overcome, integrins could open up a new range of therapeutic possibilities in oncologic treatment. This step forwards could be brought about by new approaches towards integrin inhibitors, such as combined therapies [146] and the knowledge gain through results of evolving trials and evaluations. Ongoing and future clinical trials will decide whether integrin antagonists make their way into clinical use or not.

It still remains to be determined whether targeting integrins may bring us one step closer to defeating metastatic spread, one of the biggest dilemmas in advanced cancer stages, and whether these adhesion molecules have the potential for a major breakthrough in oncologic treatment.

## Abbreviations

ADAM: A disintegrin and metalloproteinase
ADMIDAS: Adjacent to MIDAS
CAM: Cell adhesion molecuIe
CEA: Carcinoembryonic antigen
CLA: Cutaneous lymphocyte antigen
FDA: U.S. Food and Drug Administration
EMT: Epithelial mesenchymal transition
ERK: Extracellular signal-regulated kinase
ESCC: Esophageal squamous cell carcinoma
GPNMB: Glycoprotein nmb-mediated
HCELL: Hematopoietic cell E−/L-selectin ligand
HEV: High endothelial venules
IAC: Integrin adhesion complex
ICAM: Intercellular adhesion molecule
JAM-A, B, C: Junctional adhesion molecule A, B, C
LFA-1: Lymphocyte function associated antigen 1
L1-CAM: Neuronal cell adhesion molecule
MET: Mesenchymal-epithelial transition
MIDAS: Metal-ion-dependent adhesion site
PCI: Percutaneous coronary intervention
PDAC: Pancreatic ductal adenocarcinoma
PMN: Polymorphonuclear neutrophils
PSA: Prostatic specific antigen
PSGL-1: P-selectin glycoprotein ligand-1
TCF: T-cell factor
VCAM-1: Vascular cellular adhesion molecule
VLA-4: Very late antigen 4
5-FU: 5-fluorouracil
